# Trajectory and cost-effectiveness of a disability assessment tool based on the international classification of functioning, disability and health for rehabilitation of stroke patients: a retrospective cohort analysis

**DOI:** 10.3389/fneur.2025.1695706

**Published:** 2025-12-15

**Authors:** Xia Zhang, Si-Jing Chen, Er-Li Mao, Jian-Feng Luo, Xiao-Chen Fan, Jia-Li Liu, Shou-Guo Liu, Jia Guo, Rui-Ling Xu, Guang Li, Yu-Tong Zhang, Ling-Ye Zhang

**Affiliations:** 1 TCM Rehabilitation Department, Honghui Hospital, Xi'an Jiaotong University, Xi'an, China; 2 School of Rehabilitation Medicine, Nanjing Medical University, Nanjing, China; 3 Center of Rehabilitation Medicine, The First Affiliated Hospital of Nanjing Medical University, Nanjing, China; 4 Department of Biostatistics, School of Public Health, Fudan University, Shanghai, China; 5 NHC Key Laboratory of Health Technology Assessment, Fudan University, Shanghai, China; 6 Key Laboratory of Public Health Safety of Ministry of Education, Fudan University, Shanghai, China; 7 Xianxia Street Community Health Service Center, Shanghai, China; 8 Department of Neurosurgery, Xi'an Children's Hospital, Xi'an, China

**Keywords:** cost-effectiveness, group-based trajectory modeling, rehabilitation efficacy, stroke, the international classification of functioning, disability and health

## Abstract

**Background:**

How to effectively quantify and enhance the rehabilitation progress and treatment efficacy of stroke patients, as well as reduce the economic burden on patients, is of particular significance. We employed the International Classification of Functioning, Disability and Health(ICF) to assess and analyze the functional improvement of stroke inpatients during rehabilitation, to investigate the trend of rehabilitation outcome and its influencing factors in patients with different admission functional statuses, and to explore the relationship between rehabilitation efficacy and cost-effectiveness.

**Methods:**

The ICF Disability Assessment Tool was utilized to gather clinical functional data of patients at different stages of rehabilitation. Group-based trajectory modeling(GBTM) was adopted to identify the trajectories of ICF total scores, and logistic regression was applied to explore the specific factors affecting the grouping of rehabilitation trajectories, and the dysfunction, functional improvement, and cost of rehabilitation treatment in different subgroups were also comparatively analyzed.

**Results:**

A total of 95 stroke patients were included in this study. GBTM analyses generated 4 distinct ICF trajectories, namely the mild dysfunction group (17.89%), the moderate dysfunction group (35.79%), the severe dysfunction group (27.37%), and the extremely severe dysfunction group (18.95%). Patients who were older, utilized walking aids upon admission, and had dysphagia were more prone to possess the trajectory characteristics of extremely severe dysfunction. The average daily improvement in total ICF disability score decreased with the increase in disability. The rehabilitation costs were the highest in the extremely severe dysfunction group, followed by the mild dysfunction group, and the lowest in the moderate dysfunction group.

**Conclusion:**

The group-based trajectory modeling disclosed that disparities existed in functional recovery among stroke patients with varying degrees of dysfunction, with those who were older, utilized walking aids upon admission, and had dysphagia exhibited a slower recovery. Rehabilitation can improve the functional status of stroke patients, but its cost-effectiveness varies depending on the severity of dysfunction.

## Introduction

Stroke is a cerebrovascular disease with high morbidity, mortality, and disability, being the second leading cause of death globally and the third leading cause of disability-related deaths (1). In the past 30 years, the total number of stroke patients and those with disabilities has nearly doubled, and the onset of stroke is showing a trend of younger age (2). Currently, the World Health Organization estimates that the expenses paid for cerebrovascular diseases in China will rank first in the world (3). The costs of stroke include not only direct medical expenses, covering hospitalization, outpatient care, and home care; but also direct non-medical expenses, such as additional costs for social services, transportation, and nutrition; as well as indirect costs arising from productivity loss, cognitive or physical disabilities, and even death (4). The resulting increase in financial pressure on society has become a major social issue that urgently needs to be addressed.

Evidence-based medicine confirms that stroke rehabilitation is the most effective measure to reduce disability rates (5). The fundamental purpose of stroke rehabilitation is to prevent complications, improve function, and enhance daily living abilities. It is crucial to develop standardized and personalized rehabilitation plans for reducing the disability rate of patients with acute cerebrovascular disease and significantly improving their quality of life.

Functional assessment is the core of rehabilitation process. Through quantitative assessment, we can understand the patient’s functional status and the progress of treatment during rehabilitation, providing important evidence for developing personalized rehabilitation plans that meet the patient’s needs. The International Classification of Functioning, Disability and Health (ICF) is a standardized common language for evaluating function, promulgated by the World Health Organization in 2001. It reflects a system for classifying functions and assessing outcomes (6), and can serve as a clinical tool for rehabilitation assessment (7). The ICF Research Center has already developed the ICF Core Sets, the ICF Generic Set, and the ICF Rehabilitation Set. The ICF Core Sets is only targeted at specific diseases, injuries or health issues and lacks universality; the ICF Rehabilitation Set has 30 assessment items, which take a long time to evaluate, and some items are difficult to apply to the patient in hospitalization; the ICF Generic Set is overly simplistic and cannot comprehensively reflect the functional status of hospitalized patients.

Our research team previously referenced the World Health Organization’s World Health Survey, the Functional Independence Measure (8), the ICF Generic Set (9), and the Chinese Practical Assessment Criteria for Persons with Disabilities. We invited experts from various fields such as rehabilitation, nursing, and insurance to participate in multiple rounds of expert consensus and validation meetings, ultimately selecting 20 ICF items suitable for disability assessment. They include: d450 Walking, d455 Moving around, b455 Exercise tolerance functions, b525 Defecation functions, b620 Urination functions, d530 Toileting, d230 Carrying out daily routine, d510 Washing oneself, d520 Caring for body parts, d540 Dressing, d550 Eating, b130 Energy and drive functions, b134 Sleep functions, b152 Emotional functions, b280 Sensation of pain, b114 Orientation functions, b144 Memory functions, b210 Seeing functions, b230 Hearing functions, d710 Basic interpersonal interactions.

The assessment tool has good reliability and validity and can be used for functional evaluation of various conditions leading to disability, including stroke (10–12). During the assessment process, a Numerical Rating Scale is used to score the items on a scale from 0 to 10, where 0 indicates no problems at all and 10 indicates complete dysfunction. Among them, the four items like b130 Energy and drive functions, b134 Sleep functions, b152 Emotional functions, and b280 Sensation of pain are primarily in the direction of somatic functioning in ICF and are associated with the patient’s subjective feelings. These items are self-reported; for patients unable to express, their caregivers will interpret the current status of bodily functions, after which the assessor scores them. The remaining items are other-rated, scored by the evaluator based on the patient’s functional status.

Stroke often leads to varying degrees of functional impairment, affecting patients’ quality of life and resulting in significant social healthcare expenditures. Therefore, it is particularly important to effectively improve the rehabilitation progress and treatment of stroke patients. This study retrospectively analyzed the application of disability assessment tools in the rehabilitation treatment process of stroke patients, aiming to explore the application value of the assessment tool in the functional rehabilitation of stroke patients, identify the functional improvement trajectories of patients with different functional impairments and their influencing factors, and investigate the relationship between rehabilitation efficacy and cost-effectiveness. It will lay the foundation for constructing a quality control system for stroke rehabilitation in the subsequent stage and actively promote the optimization and improvement of clinical work in stroke rehabilitation.

## Materials and methods

### Study cohort

This study has been approved by the Ethics Committee of Jiangsu Provincial People’s Hospital (Approval Number: 2020-SR-148).

*Inclusion criteria*: (1) Age ≥ 18 years; (2) Clear disease diagnosis, meeting the diagnostic criteria for stroke; (3) At least two assessments by the ICF Disability Assessment Tool during hospitalization, with complete assessments at admission and the study endpoint (discharge, referral, death, or the end of study); (4) Repeated admissions were collected only for the first time.

*Exclusion criteria*: (1) Critical patients whose vital signs have not been stabilized; (2) Unable to complete the ICF Disability assessment.

This study is a retrospective cohort analysis of clinical data from stroke patients hospitalized at Jiangsu Zhongshan Geriatric Rehabilitation Hospital between January 2020 and June 2021. This hospital is a specialized rehabilitation center focusing primarily on the rehabilitation of patients with spinal cord injuries, strokes, and musculoskeletal disorders. The main medical quality control system of the hospital and the admission and discharge criteria are based on whether the patients achieve rehabilitation goals, and there is no special restriction on the length of a single hospitalization. In this study, the sample size range was determined based on the 17 independent variables, which was 5–10 times the number of independent variables (13), and considering a 10% dropout rate, the final sample size was determined to be 94–188 cases. According to the inclusion and exclusion criteria, 95 patients who met the above standards were included.

### Data collection

General information, rehabilitation costs (excluding bed fees, nursing fees, medication fees, et al.), and the scores of the ICF Disability Assessment Tool at admission, during hospitalization (re-evaluated every 2 weeks), and at discharge were collected. The ICF Disability Assessment Tool was conducted by registered nurses with standardized training and more than 3 years of experience. Informed consent was obtained from the patient and their families before the assessment started.

### Statistical analysis

Group-based trajectory modeling (GBTM) is a longitudinal data analysis technique based on the heterogeneity of overall data. Through the analysis of repeated follow-up measurements of the same individual at multiple time points, it aims to identify groups of people who follow similar trajectories on a single indicator. Statistical analysis was performed using SAS 9.4 software package. In the trajectory analysis, for the ICF disability scores as continuous data, a censored normal (CNORM) trajectory was fitted, determining the optimal number of groups.

In our study, the fitting basis of ICF trajectory grouping based on GBTM model is mainly determined by the following indicators: (1) Bayesian information criterion (BIC) values closer to 0 indicate a better fitting effect; (2) Average post-test grouping probability (Avepp) reflects the post-test probability of each individual being classified into the corresponding trajectory subgroup, with >0.7 typically used as the acceptance criterion; (3) ≥ 5% of patients are classified in each trajectory (14–16).

In the analysis of factors influencing the grouping of ICF trajectories, statistically significant univariate variables (*p* < 0.05) were included in a multivariate ordinal logit regression for variable selection. Simultaneously, a descriptive analysis of the functional impairment, functional improvement, and rehabilitation therapy costs of different groups was conducted, and the effect index, efficiency index, and benefit index were calculated to assess the cost-effectiveness of the rehabilitation treatment cost of patients. *p*-values ≤0.05 (two-sided) denoted statistically significant differences.

## Results

### Dysfunction and improvement

A total of 95 stroke patients were ultimately included in this study, encompassing 47 patients with cerebral infarction and 48 patients with cerebral hemorrhage, with an average age of 56.28 ± 16.47 years, an average length of hospital stay was 64.21 ± 52.49 days, an average modified Barthel index upon admission of 49.06 ± 27.84, an average ICF admission score of 73.52 ± 32.93, and a discharge score of 50.35 ± 27.76. By statistically analyzing the dysfunction of stroke patients in diverse items and the improvement following rehabilitation treatment, it was discovered that the majority of the patients’ dysfunction was concentrated in the functional areas of ‘self-care and activities, emotion and spirit’. Over 50% of the patients had dysfunction in items d450 (walking), d455 (Moving around), b455 (Exercise tolerance functions), d530 (toileting), d230 (Carrying out daily routine), d510 (Washing oneself), d520 (Caring for body parts), d540 (dressing), b130 (Energy and drive functions), and b152 (Emotional functions). After rehabilitation therapy, most patients with dysfunction could obtain corresponding improvement, with the highest percentage of patients improved observed in item d550 (eating), approximately 91.11%, and the average score improvement rate was the highest at 58.04%. Nevertheless, more than half of the patients demonstrated little or no improvement in d455 Moving around, b144 Memory functions, b210 Seeing functions, and b230 Hearing functions. The details of each item are shown in Table 1.

**Table 1:** Patients’ dysfunction and improvement in 20 items.

Functional area	Number	Item name	Number of dysfunction, No.	Number of improvement, No.	Admission score	Discharge score	Improvement score	Improvement rate
Self-care and activities	1	d450 Walking	94 (98.95%)	77 (81.91%)	7.38	4.82	2.56	34.69%
	2	d455 Moving around	83 (87.37%)	20 (24.10%)	8.05	7.28	0.77	9.57%
	3	b455 Exercise tolerance functions	86 (90.53%)	71 (82.56%)	4.14	1.97	2.17	52.42%
	4	b525 Defecation functions	44 (46.32%)	32 (72.73%)	2.27	1.21	1.06	46.70%
	5	b620 Urination functions	31 (32.63%)	25 (80.65%)	2.04	1.06	0.98	48.04%
	6	d530 Toileting	79 (83.16%)	58 (73.42%)	6.57	4.66	1.91	29.07%
	7	d230 Carrying out daily routine	90 (94.74%)	63 (70.00%)	6.91	5.01	1.89	27.35%
	8	d510 Washing oneself	92 (96.84%)	67 (72.83%)	7.39	5.58	1.81	24.50%
	9	d520 Caring for body parts	91 (95.79%)	69 (75.82%)	7.35	5.42	1.93	26.26%
	10	d540 Dressing	87 (91.58%)	72 (82.76%)	7.07	4.83	2.24	31.68%
	11	d550 Eating	45 (47.37%)	41 (91.11%)	2.86	1.20	1.66	58.04%
Cognition and perception	12	b114 Orientation functions	36 (37.89%)	18 (50.00%)	1.66	1.19	0.47	28.31%
	13	b144 Memory functions	43 (45.26%)	16 (37.21%)	1.54	1.16	0.38	24.68%
	14	b210 Seeing functions	33 (34.73%)	1 (3.03%)	0.92	0.89	0.02	2.17%
	15	b230 Hearing functions	6 (6.32%)	0 (0.00%)	0.20	0.20	0	0.00%
	16	d710 Basic interpersonal interactions	40 (42.11%)	29 (72.50%)	1.98	1.31	0.67	33.84%
Emotion and spirit	17	b130 Energy and drive functions	57 (60.00%)	42 (73.68%)	1.53	0.68	0.85	55.56%
	18	b152 Emotional functions	53 (55.79%)	34 (65.38%)	1.27	0.73	0.54	42.52%
	19	b280 Sensation of pain	41 (43.16%)	27 (64.29%)	1.06	0.55	0.51	48.11%
	20	b134 Sleep functions	44 (46.32%)	34 (75.56%)	1.38	0.59	0.79	57.25%

Quantitative data are presented as mean unless otherwise stated. Improvement rate = Improvement score/Admission score.

### Characterization of ICF disability score trajectories

**Figure 1:**
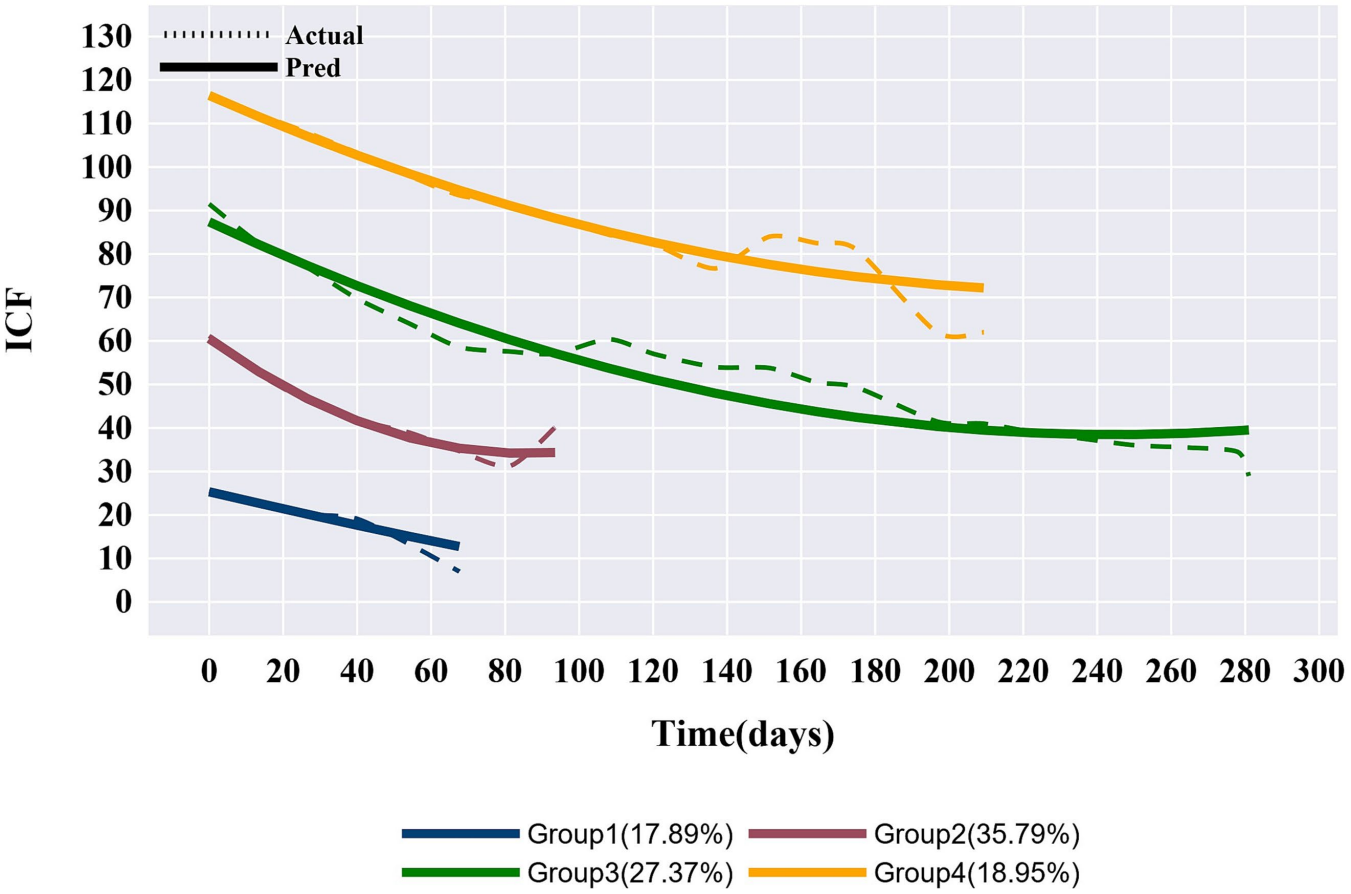
ICF trajectory group characteristics in total cohort.

GBTM was fitted according to the total ICF disability score of the longitudinal cohort population, and the optimal trajectory was ultimately identified to be 4 groups based on Avepp, BIC, the actual recovery of dysfunction, and the principle of a simplified model. In each group, the overall ICF score showed a downward trend over time, indicating that the functional status of each group improved with the increase of treatment time of patients. The four trajectory groups were as follows:

Group 1 (*n* = 17, 17.89%): The score of patients was approximately 25 at admission and decreased to approximately 12 after about 68 days of rehabilitation treatment, with a stable average improvement rate, defining as the ‘Mild dysfunction group’.
Group 2 (*n* = 34, 35.79%): The score of patients was approximately 61 at admission and decreased to approximately 35 after about 93 days of rehabilitation treatment, defining as the ‘Moderate dysfunction group’. The improvement rate was faster than the other three groups during the first 60 days, but slowed down in the later stages.
Group 3 (*n* = 26, 27.37%): The score of patients was approximately 88 at admission and decreased to approximately 40 after about 280 days of rehabilitation treatment, defining as the ‘Severe dysfunction group’. The function improved by approximately 30 points after about the first 100 days, with a faster improvement, but a slower improvement rate in the later stages.
Group 4 (*n* = 18, 18.95%): The score of patients was approximately 116 at admission and decreased to approximately 82 after the initial 120 days of rehabilitation treatment, which improved very slowly for the subsequent 90 days, by approximately 10 points, defining as the ‘Extremely severe dysfunction group’. The ICF disability score trajectories of the four groups of patients are shown in Figure 1.

### Factors influencing trajectory grouping

The grouping of ICF disability score trajectory was employed as the dependent variable, and the independent variables encompassed gender, age, education level, *et al*. Univariate analysis revealed that in terms of gender, males outnumbered females in all groups; regarding age, the average age of four groups was 45.4 ± 15.7 years, 53.2 ± 16.0 years, 63.7 ± 15.4 years, and 61.8 ± 13.0 years respectively, with the severe and extremely severe dysfunction groups having a higher average age. In terms of dysphagia, the proportion of individuals with swallowing disorders in four groups was 5.88, 11.76, 34.62, and 44.44%, respectively, with the proportion of dysphagia in the extremely severe dysfunction group was the highest. In terms of cognitive function, there was no cognitive impairment in the mild dysfunction group, 4/34 in the moderate dysfunction group, 12/26 in the severe dysfunction group, and 12/18 in the extremely severe dysfunction group, indicating a higher proportion of cognitive impairment in the severe and extremely severe dysfunction groups. In the use of urine bags or diapers, the proportion of the extremely severe dysfunction group was the highest at 50%. At the time of admission, the proportion of patients needing assistive devices for walking in the moderate, severe, and extremely severe dysfunction groups was higher than those in the mild dysfunction group. Single factor analysis of each variable is shown in Table 2.

**Table 2:** Univariate analysis by ICF disability score trajectory.

Variables	ICF disability score				Statistics	*p*
	Group 1	Group 2	Group 3	Group 4		
Disease course [days, median (IQ)]	86 (54, 310)	66 (35, 130)	56 (23, 137)	84.5 (35, 189)	*H* = 2.88	0.41^#^
Gender					–	0.008*
Male	14 (82.35)	29 (85.29)	12 (46.15)	14 (77.78)		
Female	3 (17.65)	5 (14.71)	14 (53.85)	4 (22.22)		
Age (years, x̄±S)	45.4 ± 15.7	53.2 ± 16.0	63.7 ± 15.4	61.8 ± 13.0	*F* = 6.14	<0.001
Hypertension					–	0.961*
Yes	14 (82.35)	25 (73.53)	20 (76.92)	14 (77.78)		
No	3 (17.65)	9 (26.47)	6 (23.08)	4 (22.22)		
Diabetes					–	1.000*
Yes	3 (17.65)	7 (20.59)	6 (23.08)	4 (22.22)		
No	14 (82.35)	27 (79.41)	20 (76.92)	14 (77.78)		
Education level					–	0.086*
Illiterate	1 (5.88)	0 (0.00)	2 (7.69)	0 (0.00)		
Primary school	0 (0.00)	2 (5.88)	5 (19.23)	2 (11.11)		
Secondary Technical school	3 (17.65)	8 (23.53)	9 (34.62)	7 (38.89)		
College or above	13 (76.47)	24 (70.59)	10 (38.46)	9 (50.00)		
Diagnosis					*χ^2^* = 4.39	0.222
Cerebral hemorrhage	11 (64.71)	19 (55.88)	9 (34.62)	9 (50.00)		
Cerebral infarction	6 (35.29)	15 (44.12)	17 (65.38)	9 (50.00)		
Emotional disorders					–	0.591*
Yes	1 (5.88)	4 (11.76)	3 (11.54)	4 (22.22)		
No	16 (94.12)	30 (88.24)	23 (88.46)	14 (77.78)		
Shoulder pain					–	0.692*
Yes	0 (0.00)	3(8.82)	2(7.69)	2(11.11)		
No	17 (100.00)	31 (91.18)	24 (92.31)	16 (88.89)		
Dysphagia					–	0.008*
Yes	1 (5.88)	4 (11.76)	9 (34.62)	8 (44.44)		
No	16 (94.12)	30 (88.24)	17 (65.38)	10 (55.56)		
Use of urine bags or diapers					–	<0.001*
Yes	0 (0.00)	3 (8.82)	4 (15.38)	9 (50.00)		
No	17 (100.00)	31 (91.18)	22 (84.62)	9 (50.00)		
Need for assistive devices					–	<0.001*
Yes	7 (41.18)	25 (73.53)	25 (96.15)	16 (88.89)		
No	10 (58.82)	9 (26.47)	1 (3.85)	2 (11.11)		
Surgery					*χ^2^* = 3.10	0.377
Yes	5 (29.41)	10 (29.41)	7 (26.92)	9 (50.00)		
No	12 (70.59)	24 (70.59)	19 (73.08)	9 (50.00)		
Ataxia					–	0.458*
Yes	2 (11.76)	1 (2.94)	3 (11.54)	2 (11.11)		
No	15 (88.24)	33 (97.06)	23 (88.46)	16 (88.89)		
Hemiplegic side					–	0.692*
Left	6 (35.29)	17 (50.00)	14 (53.85)	9 (50.00)		
Right	9 (52.94)	14 (41.18)	7 (26.92)	7 (38.89)		
Bilateral	2 (11.76)	3 (8.82)	5 (19.23)	2 (11.11)		
Cognitive impairment					*χ^2^* = 27.69	<0.001
Yes	0 (0.00)	4(11.76)	12(46.15)	12(66.67)		
No	17 (100.00)	30 (88.24)	14 (53.85)	6 (33.33)		
Aphasia					–	0.697*
Yes	2 (11.76)	4 (11.76)	5 (19.23)	4 (22.22)		
No	15 (88.24)	30 (88.24)	21 (80.77)	14 (77.78)		

‘*’, Fisher’s exact test; ‘#’, Wilcoxon rank sum test; ‘–’, Unacquired. Continuous data are presented as median (interquartile) or mean ± standard deviation, whereas categorical data are presented as *n* (%).

Variables with *p* ≤ 0.05 from the univariate analysis were incorporated as independent variables in the ordinal logit regression for analysis, and the age was included as a continuous variable, while other independent variables were included in the model as categorical variables. Considering the significance of surgery for the actual clinical context, it was included in the regression model despite it’s unsatisfactory results in the univariate analysis. Taking the mild dysfunction group as the reference, the results indicated that the model likelihood ratio *χ*^2^ was 70.68 (*p* < 0.0001), indicating that the model construction was statistically significant. Simultaneously, the model conformed to the principle of parallel line assumption (*χ*^2^ = 20.11, *p* = 0.127 > 0.05); the bias and Pearson goodness-of-fit results *χ*^2^ were 170.05 and 211.52 respectively, with *p* = 1.00 and 0.97, both of which were greater than 0.05, indicating that the model fitted the data well. It can be observed that stroke patients who are older, use assistive devices for walking upon admission, and have dysphagia are more likely to exhibit characteristics of extremely severe dysfunction trajectories. Multivariate analysis of significant Variables is shown in Table 3.

**Table 3:** Multivariate ordinal logit regression analysis of significant variables.

Variables	Parameter estimation	Standard error	Wald *𝜒*^2^	*p*	OR (95%CI)
Intercept	−11.20	3.386	10.594	0.001	
Gender					
Male					1.00 (ref)
Female	−0.404	1.247	0.105	0.746	0.67 (0.06–7.69)
Dysphagia					
No					1.00 (ref)
Yes	3.180	1.477	4.635	0.031	24.05 (1.33–435.03)
Use of urine bags or diapers					
No					1.00 (ref)
Yes	12.917	239.84	0.003	0.957	407,343 (0.00-57E208)
Need for assistive devices					
No					1.00 (ref)
Yes	3.108	1.386	5.031	0.025	22.37 (1.48–338.19)
Surgery					
No					1.00 (ref)
Yes	2.159	1.285	2.822	0.093	8.66 (0.70–107.44)
Cognitive impairment					
No					1.00 (ref)
Yes	14.345	178.17	0.006	0.936	1.7E6 (0.00–78E156)
Age (years)	0.109	0.045	5.857	0.016	1.12 (1.02–1.22)

### Rehabilitation efficacy and cost-effectiveness

The total ICF scores, the length of hospital stay, and the cost of inpatient rehabilitation treatment of four groups were statistically analyzed, as shown in Table 4. The mild dysfunction group had the most improvement per day, and the extremely severe dysfunction group spent the most on rehabilitation treatment for each 1-point improvement.

**Table 4:** Functional improvement, length of hospital stay and expenses in four groups.

Variables	Group 1	Group 2	Group 3	Group 4
Admission score	25.2 ± 12.4	61.6 ± 14.6	91.3 ± 13.5	115.9 ± 13.0
Discharge score	16.6 ± 9.6	38.9 ± 10.2	59.8 ± 16.2	90.1 ± 19.5
Improvement score	8.5 ± 9.3	22.8 ± 17.8	31.5 ± 17.7	25.9 ± 17.3
Admission MBI score	84.7 ± 11.0	59.4 ± 20.0	32.4 ± 17.0	20.0 ± 12.9
Length of hospital stay	31.9 ± 18.1	49.4 ± 27.6	87.6 ± 74.0	88.9 ± 49.4
Treatment costs	15,463 ± 11,193	26,425 ± 16,128	47,840 ± 46,622	56,321 ± 37,624
Daily treatment cost	484.73	531.28	546.12	633.53
Effect index	34.13%	36.85%	34.5%	22.26%
Efficiency index	1.07%	0.75%	0.39%	0.25%
Benefit index	1819.18	1158.99	1518.70	2174.56

Effect index = (Admission score − Discharge score)/Admission score; Efficiency index = Effect index/Length of hospital stay; Benefit index = Treatment costs/Improvement score.

## Discussion

As the aging population continues to grow, existing medical resources will find it challenging to handle the escalating number of individuals with disabilities resulting from stroke. Effective and standardized rehabilitation treatment can alleviate the economic burden of the disease for both patients and society. The ICF adopts a ‘bio-psycho-social’ medical model to comprehensively assess human dysfunction and the rehabilitation process, which not only focuses on the biological damage to the organization and organs of disabled individuals but also emphasizes the impact of psychological and social factors. It constructs a new model encompassing health, function, and disability, providing a novel and practical methodology for the diagnosis and treatment strategies, efficacy evaluation, and clinical research of stroke rehabilitation (17), and it’s effect is superior to that of the traditional routine rehabilitation training.

This study collected data of the ICF disability score of stroke patients during inpatient rehabilitation. By analyzing the number and improvement of individuals with dysfunction in 20 items, it was found that over 80% of patients exhibited dysfunction in the domain of ‘self-care and activities’, specifically concentrated in items d450 Walking, d455 Moving around, b455 Exercise tolerance functions, d530 Toileting, d230 Carrying out daily routine, d510 Washing oneself, d520 Caring for body parts, and d540 Dressing, which aligns with clinical realities, and these items also the focus of evaluation and treatment in clinical practice (18). Additionally, over 50% of patients exhibit disorders in b130 Energy and drive functions and b152 Emotional functions, which might be associated with post-stroke depression. Studies have indicated that stroke is one of the principal causes of depression and dementia (2). Therefore, during the rehabilitation treatment process, it is essential to not only focus on the recovery of patients’ motor functions but also strengthen their psychological treatment.

The dysfunction in all fields improved after rehabilitation treatment, yet there were disparities in the number and degree of improvement in each item. More than half of patients showed no improvement in d455 Moving around, b144 Memory functions, b210 Seeing functions, and b230 Hearing functions, and even the low average score was found in rest patients. On the one hand, it is challenging to enhance the function corresponding to these items, requiring a considerable amount of time; on the other hand, as these items are not commonly regarded as rehabilitation goals, there are few rehabilitation trainings in these directions. Therefore, the screening of assessment items and the content and form of assessment need to be further refined in the future. The improvement rates of b130 Energy and drive functions, b152 Emotional functions, b280 Sensation of pain, and b134 Sleep functions were relatively high, possibly because of the fact that they were self-evaluation items, related to the emotional and cognitive state of patients (19) and were greatly influenced by the patient’s subjective willingness. Hence, during the process of exercise rehabilitation, it is also essential to conduct psychological rehabilitation for patients, guiding them to identify and modify negative thoughts, cognition, and feelings, establish new cognition, promote the increase of patients’ subjective initiative, and thereby enhance the rehabilitation effect (20).

The GBTM is mainly employed for longitudinal data with overall heterogeneity, exploring and identifying potential subgroups with distinct development trajectories within the population, while individuals within the same subgroup share the same developmental trend (21). Currently, it is commonly utilized in fields such as psychology, biology, and behavioral science, and yet rarely applied in the field of rehabilitation medicine (22–24). This study was the first to employ the GBTM to model the ICF Disability Set over the course of rehabilitation and identified four trajectory subgroups: mild dysfunction group, moderate dysfunction group, severe dysfunction group, and extremely severe dysfunction group, each with different developmental patterns. Patients in the mild dysfunction group demonstrated a rapider functional advancement and a shorter hospitalization duration. In the moderate dysfunction group, the functional enhancement was conspicuous during the initial 60 days of treatment, yet decelerated in the subsequent period. In the severe dysfunction group, the functional improvement was more pronounced within the first 100 days of treatment and reached a plateau after 180 days. Patients in the extremely severe dysfunction group manifested rapid functional improvement within the first 120 days of treatment, but the functional improvement was sluggish in the later stage. It can be perceived that the functional status of patients encompasses a period of rapid functional recovery and a plateau period, even in the context of hospitalization. This is consistent with the majority of publications indicating that functional recovery in stroke patients stabilizes between 3 and 6 months post-onset (25). A stroke study conducted in Copenhagen demonstrated that the rate of functional recovery after stroke is closely associated with the initial stroke severity; the greater the severity, the longer the time required to reach optimal ADL function. Even in patients with severe and very severe strokes, significant neurological and functional recovery should not be anticipated after the first 5 months post-stroke (26). Other studies have also reported that stroke patients show minimal functional improvement after 12 to 14 weeks post-onset (27, 28). This phenomenon is associated with slower neurological recovery in patients during the later stages. Additionally, prolonged inability to perform activities in daily living can lead to demotivation, which in turn affects functional recovery (29).

The analysis of the clinical data of these four groups discovered that stroke patients who were older, utilized walking aids upon admission, and had dysphagia were more prone to possess the trajectory characteristics of extremely severe dysfunction. So, we should pay more attention to these patients and adopt effective rehabilitation measures to facilitate functional recovery. The functional recovery scores of elderly stroke patients in daily activities, moving, and participation were significantly lower than those of younger patients (30). Therefore, rehabilitation goals and expectations for elderly patients need to be more realistic and individualized, which should focus more on improving the quality of life, reducing complications, and prolonging survival time, rather than achieving a full functional recovery. Regarding the use of assistive devices, it is necessary to popularizing their knowledge to patients and their families to improve their ability to use in a correct way, thereby avoiding disuse paralysis resulting from inappropriate use of assistive devices, or secondary injuries caused by safety issues, which will exacerbate the dysfunction of patients (31). Dysphagia prevents patients from eating normally, leading to malnutrition. Therefore, all stroke patients should undergo a comprehensive assessment of swallowing function and nutritional status early on, and comprehensive rehabilitation strategies and measures should be adopted for patients with dysphagia to improve their swallowing function. We believe that reasonable intervention of the three aforementioned influencing factors will promote the rehabilitation process of stroke patients.

Analysis of the functional improvement and treatment costs of four groups revealed that the mild dysfunction group had the highest improvement per day and the lowest daily cost, and the moderate dysfunction group exhibited the most significant functional improvement during hospitalization and the lowest cost of rehabilitation treatment for each 1-point improvement. It might be attributed to the fact that the mild dysfunction group has the least impairment, while the potential for improvement is limited. Previous studies have analyzed the rehabilitation effects and cost-effectiveness of stroke patients using the ICF Core Sets (comprehensive version), using the ranked data from 0 to 4, ultimately concluding that the average cost was 327.50 yuan per point improvement in function (32). These studies encompasses numerous items and requires a considerable amount of time for evaluation. Some items are repetitive, and certain items are not applicable to hospitalized patients, thereby increasing the difficulty of the clinical application of ICF. In this study, 20 items were employed to comprehensively assess the functional status of inpatient rehabilitation for stroke patients, which was relatively straightforward. Additionally, this study adopted a numerical assessment scale for scoring, which allowed for mathematical operations such as addition, subtraction, multiplication, and division. In this manner, the degree of functional improvement and the cost of inpatient rehabilitation could be calculated to obtain a more reliable rehabilitation effect and cost–benefit analysis, which is conducive to guiding the trend of functional improvement and medical cost of newly admitted patients. Simultaneously, this study discovered that patients with diverse functional disorders exhibit different rehabilitation cost-effectiveness. In the future, the medical insurance bureau could adopt distinct medical insurance payment approaches based on the type of dysfunction to optimize the allocation of rehabilitation medical resources.

This study also has certain limitations. Firstly, this was a single-center study with an under-representative sample, which might restrict the generalizability of the results. Secondly, the impact of the stroke stage, stroke subtype, treatment method, and the treatment timeline on functional recovery was not examined. Thirdly, the rehabilitation process for stroke is complex and influenced by a variety of factors, encompassing physiological, psychological, social, and even environmental dimensions. The clinical data of patients collected in this study are limited and fail to cover all relevant influencing factors and indicators, exerting a certain influence on the research results. Fourthly, there is limited analysis of the plateau phase during the rehabilitation process in the four groups. In the future, our group intends to broaden the investigation scope, augment the sample size, incorporate more diverse factors, and combine clinical practice with the results of this study to further improve assessment tools and data collection, continuing to explore the relationship between the recovery patterns and economic costs of patients with various functional disorders.

## Conclusion

The ICF-based disability assessment tool effectively evaluates the functional impairment in stroke patients, providing a foundation for designing personalized rehabilitation programs. The GBTM identifies heterogeneity in functional recovery among stroke patients. During rehabilitation, particular attention should be devoted to the older patient who requires assistive devices for ambulation at admission and exhibit dysphagia, with targeted efforts to improve both walking ability and swallowing function to expedite recovery. Rehabilitation can enhance the functional status of stroke patients; however, its cost-effectiveness varies according to the severity of functional impairment at admission. In the future, we intend to further expand the sample size and conduct multicenter studies to investigate the cost-effectiveness among patients with varying functional disabilities.

## Data Availability

The raw data supporting the conclusions of this article will be made available by the authors, without undue reservation.
